# Predictive value of frontal alpha asymmetry in evaluating prognosis of pediatric status epilepticus based on EEG assessment

**DOI:** 10.1097/MD.0000000000048417

**Published:** 2026-05-15

**Authors:** Liping Xie, Tao Yu, Jielan Zhou, Yaqin Zhang

**Affiliations:** a Department of Pediatrics, West China Second University Hospital, Sichuan University, Chengdu City, Sichuan Province, China; b Key Laboratory of Birth Defects and Related Diseases of Women and Children (Sichuan University), Ministry of Education, Sichuan University, Chengdu City, Sichuan Province, China.

**Keywords:** children, EEG, frontal alpha asymmetry, status epilepticus

## Abstract

To investigate the predictive value of frontal alpha asymmetry (FAA) assessed by electroencephalogram in the prognosis of pediatric status epilepticus (SE). A retrospective analysis was conducted on 177 pediatric SE patients admitted to our hospital from January 2021 to December 2024. Patients were divided into a good prognosis group (n = 126) and a poor prognosis group (n = 51) based on their clinical outcomes. Univariate and binary logistic regression analyses were performed to assess the influencing factors. The predictive value of FAA for the prognosis of pediatric status epilepticus was evaluated with receiver operating characteristic curve analysis. Significant differences were found between the 2 groups in terms of SE onset time, time from medication to seizure cessation, and FAA (*P* < .05). Binary logistics regression analysis indicated that the time from medication to seizure cessation and FAA were independent influencing factors for the prognosis of pediatric SE (*P* < .05). Receiver operating characteristic analysis revealed an area under the curve of 0.805 for FAA, with a standard error of 0.035 (95% confidence interval: 0.738–0.873), a Youden index of 0.43, a sensitivity of 90.20%, and a specificity of 52.38%. The optimal cutoff value for FAA was 88.96 (×10^‐3^ μV), with a higher probability of good prognosis in patients with FAA > 88.96 in comparison to those with FAA ≤ 88.96 (*P* < .05). Patients with FAA > 88.96 also had lower levels of glial fibrillary acidic protein, S100-β protein, and gamma-aminobutyric acid after treatment compared to those with FAA ≤ 88.96. There was a statistically significant difference (*P* < .05). FAA holds certain value in predicting the prognosis of pediatric SE and can provide reference for clinical decision-making.

## 1. Introduction

Epilepsy, as a common neurological disorder, with status epilepticus (SE) being one of the neurological emergencies, seriously threatens the life and health of patients. Electroencephalogram (EEG), as an important neurophysiological examination method, can reflect the electrical activity of brain neurons. It is a routine examination for SE, but there are limited researches on the application of related indicators in SE. Frontal alpha asymmetry (FAA), as an indicator of EEG, may be closely related to the neural function and pathological state of the brain. Previous studies^[1,2]^ have shown rapid developments in research on the relationship between frontal EEG asymmetry and emotions, personality, and neuropsychiatric disorders. FAA in the resting state, known as trait frontal alpha asymmetry, is associated with individual differences in various traits; in task conditions, known as state frontal brain alpha asymmetry, it is related to operations affecting individual emotional states.^[3]^ Previous studies have mainly focused on the functional role of frontal EEG alpha asymmetry in the resting state in patients with depression. Research in this field has provided an important perspective for understanding the relationship between brain neural activity and emotions, diseases. However, there are limited researches on this topic in pediatric SE.^[4]^ Functional abnormalities in the frontal lobe of children with SE exist, and alpha waves, as one of the basic waveforms in EEG, may contain important information related to disease prognosis in terms of their asymmetry changes.^[5,6]^ This study focuses on evaluating the predictive value of EEG-assessed FAA for the prognosis of pediatric SE, aiming to provide a more scientific and accurate reference for the prognosis assessment and treatment decisions of pediatric SE.

### 1.1. Research objects

**Figure 1.**
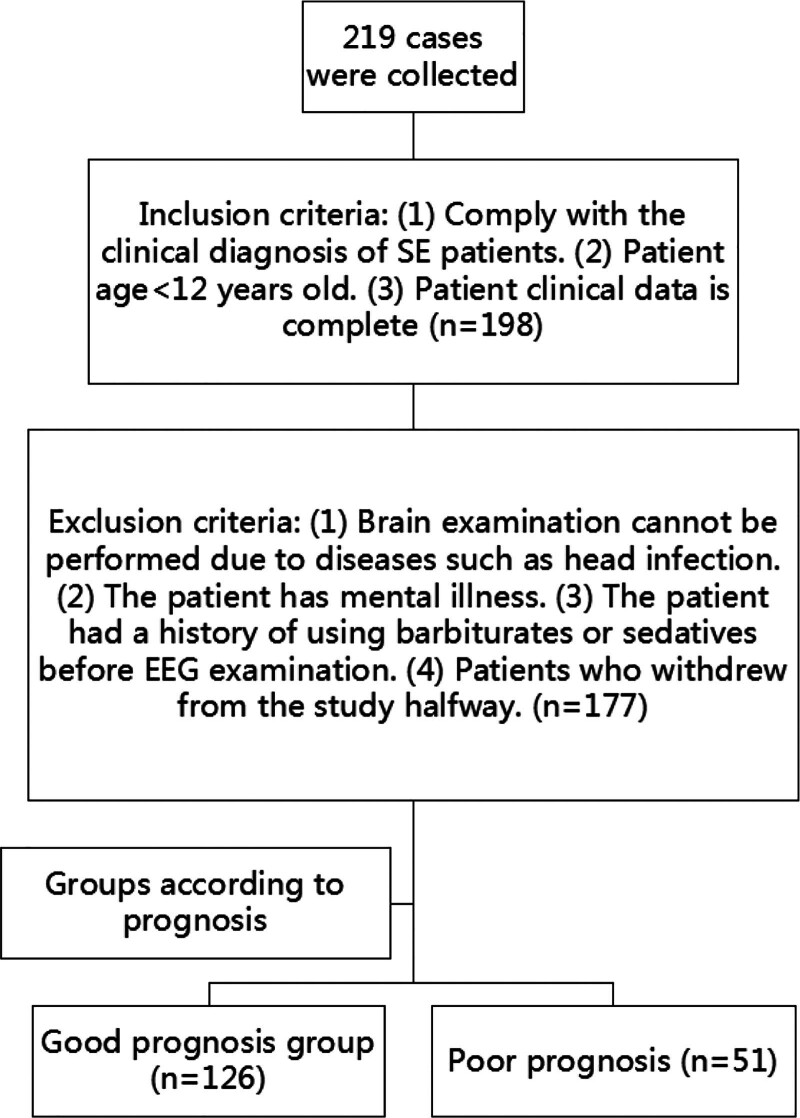
Flowchart.

*Sample size*: The sample size of this study is calculated using the overall proportion formula $N=Z_{\alpha /2}^{2}\pi 1-\pi /\delta^{2}$, where π is taken as 13.9%,^[7]^ the test level α is 0.05, and the allowable error δ is taken as 0.06. The calculated sample size is at least about 126. Taking into account the existence of invalid samples, the sample size after expanding by 20%, is at least 152. Data from 200 cases collected in this study were screened and finally 177 pediatric patients with SE admitted to our hospital from January 2021 to December 2024 were retrospectively selected. Patients were divided into a good prognosis group (n = 126) and a poor prognosis group (n = 51) based on their clinical outcomes. The inclusion criteria were as follows: all patients met the clinical diagnosis criteria for SE.^[8]^ Age < 12 years old. Patients had complete clinical data. The exclusion criteria were as follows: presence of diseases like head infections that prevented EEG examination. Patients with psychiatric disorders. History of barbiturate or sedative drug use before EEG examination. Patients who withdrew from the study prematurely (Fig. 1).

### 1.2. Research methods

#### 1.2.1. EEG data collection and analysis

This study was approved by the Ethics Committee of West China Second University Hospital. This study was designed as a single-center retrospective observational cohort study. Data from pediatric patients diagnosed with SE and admitted to our hospital between January 2021 and December 2024 were retrospectively collected and analyzed. A total of 200 cases were initially screened, and after applying the inclusion and exclusion criteria, 177 pediatric patients were ultimately included in the final analysis. Collect clinical data from electroencephalography-related examinations performed on patients during the baseline period (i.e., examinations performed at the beginning of admission). Our hospital utilized the NuAmps EEG amplifier from neuroscan along with a high-density electrode cap for the examination. The detection process involved the following steps: before the EEG measurement, participants were instructed to refrain from smoking and drinking coffee for 2 hours and from consuming alcohol for 12 hours to ensure the accuracy and stability of the EEG data. EEG recordings were conducted in a soundproof room with dim lighting and adjustable temperature to minimize external factors’ interference with the EEG signals. During the recording process, patients were instructed to undergo resting EEG recordings with eyes open and eyes closed relaxation states, each lasting for 4 minutes. High-density EEG equipment was used for data collection, following the 10 to 20 electrode placement system standardized by the International Federation of Clinical Neurophysiology. EEG signals were sampled at a rate of 500 Hz, with the reference electrode chosen as A2 and subsequently transformed into a common average reference. This study focused on electrodes in the frontal lobe (F3, F4) for analysis, ensuring electrode impedance did not exceed 5 kΩ to maintain signal quality. Additionally, eye movements were monitored with 2 pairs of additional tin electrodes for recording electrooculography. One pair of vertical eye electrodes were placed 3 mm above the center of the left eyebrow and 1.5 cm below the left lower eyelid to detect blinking and vertical eye movements. The other pair of horizontal eye electrodes were positioned 1.5 cm outside the outer corners of both eyes to monitor horizontal eye movements. The recorded data were subsequently analyzed offline. Offline analysis was performed using the EEG Laboratory Toolbox v2021.0 within the Matrix Laboratory 2019b software environment. The analysis involved manually removing noisy segments to enhance data quality, applying bandpass filtering of 0.3 to 40 Hz to retain valid EEG signal components, estimating power spectral density of the filtered EEG signals, extracting power spectral density values in the Alpha band (8–13 Hz), and calculating the mean energy of the Alpha band for each electrode to support subsequent analysis. Calculation of the FAA index: FAA = (F4 ‐ F3)/(F4 + F3).

#### 1.2.2. Prognostic assessment

The Glasgow Outcome Scale (GOS) score of the pediatric patients at discharge 1 month later was collected and utilized to evaluate the patients’ prognosis. The GOS scale is divided into 5 levels, with the specific meanings of each level as follows: Level I indicates death; Level II represents a vegetative state; Level III implies that the child is unable to live independently and has severe disabilities; Level IV indicates that the child requires supervision within 24 hours; and Level V indicates that the child can barely maintain daily life and social functions independently, with moderate disabilities, or can learn and socialize normally but may have mild disabilities. Levels I, II, III, and IV are classified as poor prognosis, while Level V is classified as a good prognosis.

#### 1.2.3. General information and laboratory data

Use electronic medical record system to collect general data of patients at admission, and pre- and posttreatment serum biomarker test results were collected. The main biomarkers collected included glial fibrillary acidic protein (GFAP), S100-β protein (μg/L), and γ-aminobutyric acid (GABA).

### 1.3. Statistical analysis

The experimental data collected were analyzed using Statistical Package for the Social Sciences 27.0 (International Business Machines Corporation, Armonk). The Shapiro–Wilk test was used for normality testing. For normally distributed metric data in the experimental data, results were expressed as $\bar{X}\pm S$. Comparisons were made with independent sample *t* tests. Count data were presented as frequencies or rates, and comparisons were conducted with χ2 test or Fisher exact test. Influencing factors were analyzed with univariate and binary logistics regression analysis. The predictive value of FAA for the prognosis of pediatric SE was evaluated using the receiver operating characteristic (ROC) curve. A significance level of *P* < .05 was considered statistically significant.

## 2. Results

### 2.1. Univariate analysis of factors influencing prognosis of pediatric status epilepticus

Statistical analysis revealed significant differences (*P* < .05) in the comparison of seizure duration, time from medication to seizure cessation, and FAA between the 2 groups of patients with SE. See Table 1 for details.

**Table 1:** Univariate analysis of factors influencing prognosis of pediatric status epilepticus.

Indicator		Good prognosis group (n = 126)	Poor prognosis group (n = 51)	*t/χ*^2^ value	*P* value
Age (yr)		6.54 ± 1.22	6.28 ± 1.26	1.272	.205
Gender	Male	54	25	0.558	.455
	Female	72	26		
BMI (kg/m^2^)		21.54 ± 1.94	21.67 ± 1.88	0.407	.684
SE duration (min)	>60	42	29	8.367	.004
	≤60	84	22		
History of epilepsy	Yes	37	17	0.270	.604
	No	89	34		
Seizure type	Convulsive	102	41	0.007	.932
	Nonconvulsive	24	10		
Time to seizure cessation with medication (min)	>30	25	19	5.894	.015
	≤30	101	32		
PICU stay (d)		6.55 ± 1.28	6.79 ± 1.08	1.179	.240
FAA (×10^‐3^ μV)		89.10 ± 17.04	66.64 ± 17.77	7.844	<.001
Underlying disease	Primary epilepsy	42	20	1.729	.421
	Viral/bacterial	39	18		
	Others	45	13		
Respiratory failure	Yes	10	6	0.647	.421
	No	116	45		
Circulation (vasopressor use)	Yes	7	6	2.057	.152
	No	119	45		

BMI = body mass index, FAA = frontal alpha asymmetry, SE = status epilepticus.

### 2.2. Binary logistics regression analysis of influencing factors

Using the significant variables identified in the univariate analysis as independent variables, and assigning values to them, an analysis was conducted with the prognosis as the dependent variable (poor prognosis = 1, good prognosis = 0). The results of the binary logistics regression analysis indicate that the time to seizure cessation with medication and FAA were independent factors associated with the prognosis of pediatric SE (*P* < .05). See Tables 2 and 3 for details.

**Table 2:** Variable assignment.

Influencing factor	Assignment
SE duration	≤60 = 0, >60 = 1
Time to seizure cessation with medication	≤30 = 0, >30 = 1
FAA	Original value

FAA = frontal alpha asymmetry, SE = status epilepticus.

**Table 3:** Binary logistics regression analysis results.

Variable	β	Standard error	Wald	*P*	Exp(β)	95% CI	
						Lower limit	Upper limit
FAA	-0.077	0.013	32.944	<.001	0.926	0.902	0.951
SE duration	0.370	0.416	0.789	.375	1.447	0.640	3.271
Time to seizure cessation with medication	1.010	0.439	5.308	.021	2.747	1.163	6.488
Constant	4.605	0.999	21.238	.000	99.961	–	–

CI = confidence interval, FAA = frontal alpha asymmetry, SE = status epilepticus.

### 2.3. ROC curve analysis of predictive value of indicators

**Figure 2.**
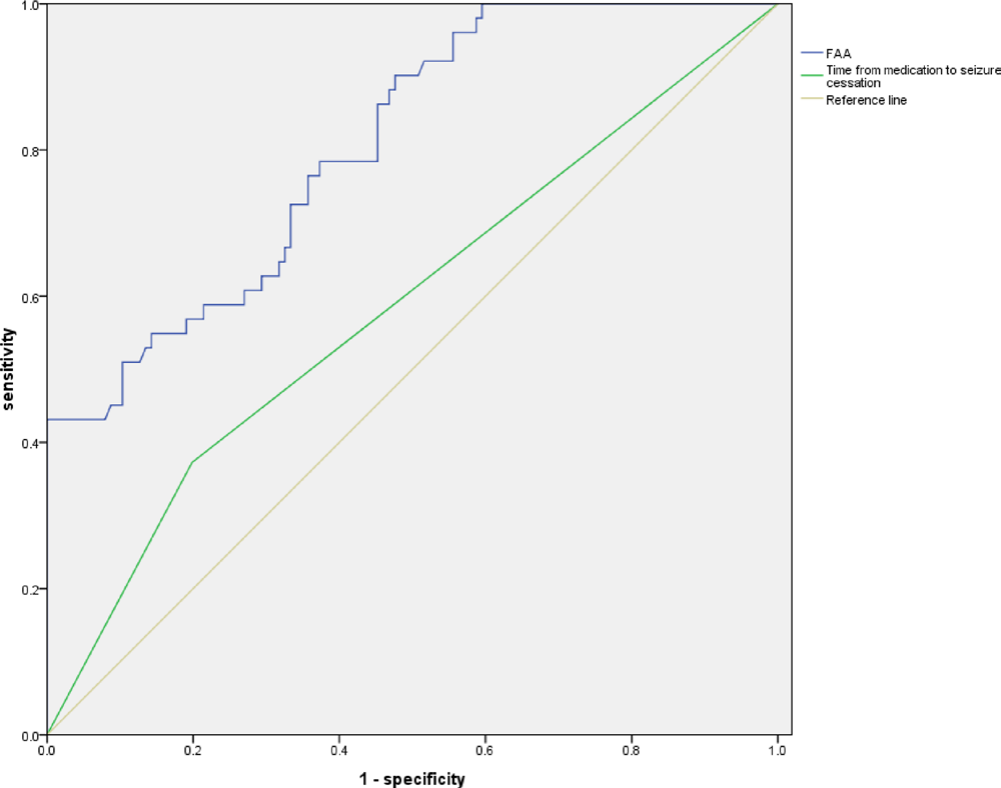
ROC curve. ROC = receiver operating characteristic.

The ROC analysis results indicated that the area under the curve for FAA was 0.805, with a standard error of 0.035 (95% confidence interval: 0.738–0.873). The Youden index was 0.43. At this point, the sensitivity was 90.20%, specificity was 52.38%, and the optimal cutoff value was 88.96 (×10^‐3^ μV), as shown in Table 4 and Figure 2.

**Table 4:** ROC analysis results.

Indicator	AUC	SE	95% CI	Youden	Sensitivity	Specificity	*P* value	Optimal cutoff value
FAA	0.805	0.035	0.738–0.873	0.43	90.20	52.38	<.001	88.96
Time to seizure cessation with medication	0.587	0.049	0.491–0.683	0.17	37.25	80.16	.070	–

AUC = area under the curve, CI = confidence interval, FAA = frontal alpha asymmetry, ROC = receiver operating characteristic, SE = status epilepticus.

### 2.4. Optimal cutoff value of FAA and clinical outcomes

The group of patients with FAA > 88.96 had a higher probability of good prognosis in comparison to the group with FAA ≤ 88.96, and the difference was statistically significant (*P* < .05), as shown in Table 5.

**Table 5:** Optimal cutoff value of FAA and clinical outcomes.

Outcome	≤88.96 (n = 106)	>88.96 (n = 71)	*Z/χ*^2^ value	*P* value
Good prognosis	60	66	–	<.001
Poor prognosis	46	5		

FAA = frontal alpha asymmetry.

### 2.5. Comparison of serum indicators before and after treatment in patients with different FAA values

Patients in the FAA > 88.96 group showed lower levels of GFAP, S100-β protein, and GABA after treatment in comparison to the FAA ≤ 88.96 group, with statistically significant differences (*P* < .05), as shown in Table 6.

**Table 6:** Comparison of serum indicators before and after treatment in patients with different FAA values.

Indicator		≤88.96 (n = 106)	>88.96 (n = 71)	*t/χ*^2^ value	*P* value
GFAP (ng/L)	Before treatment	8.93 ± 1.20	9.04 ± 1.19	0.600	.549
	After treatment	6.04 ± 1.31	5.17 ± 1.32	4.317	<.001
S100-β protein (μg/L)	Before treatment	19.28 ± 4.11	18.96 ± 4.26	0.500	.617
	After treatment	10.54 ± 2.30	8.32 ± 1.89	6.747	<.001
GABA (μmol/L)	Before treatment	69.88 ± 10.41	70.09 ± 10.55	0.131	.896
	After treatment	61.87 ± 7.14	54.35 ± 7.37	6.780	<.001

FAA = frontal alpha asymmetry, GABA = gamma-aminobutyric acid, GFAP = glial fibrillary acidic protein.

## 3. Discussion

SE has always been a focal point of clinical attention regarding pediatric neurological emergencies. While traditional assessment methods offer valuable insights, they come with limitations. This study retrospectively analyzed 177 cases of pediatric SE patients, revealing the significant value of FAA in predicting the prognosis of pediatric SE.

The study results indicated that, through univariate and binary logistics regression analyses, the duration from medication administration to seizure cessation and FAA were independent factors influencing the prognosis of pediatric SE. Further ROC analysis revealed a low predictive value (*P* = .070) for the duration from medication administration to seizure cessation, whereas FAA demonstrated an area under the curve of 0.805, showing high specificity and sensitivity. Analyzing these results, the frontal lobe plays a crucial role in the brain’s electrical activity, with FAA reflecting the difference in alpha wave power between the left and right frontal lobes. Alpha waves are typically associated with the brain’s inhibitory state, and changes in their power may reflect the excitability levels of neurons.^[9]^ In SE, abnormal neuron excitability is a key mechanism of seizures. An abnormal FAA may signify an imbalance in the excitability of frontal lobe neurons. When the power of alpha waves decreases in one frontal lobe, it may indicate increased excitability of neurons in that area, making seizure occurrence more likely. This imbalance in excitability could disrupt the brain’s normal neural regulatory mechanisms, making seizures difficult to control and affecting prognosis. In pediatric SE patients, an abnormal FAA may reflect similar disruptions in neuronal excitability regulation, subsequently impacting disease prognosis. Additionally, normal brain function relies on synchronized activity between neurons. The generation of α waves is closely related to the synchronicity of neural networks in the frontal lobes. An abnormal FAA may reflect changes in the synchronicity of frontal lobe neural networks. Previous studies, such as those by Fitzgerald PJ et al,^[6]^ have also shown the regulatory role of neurotransmitters, further supporting this role. In SE, abnormal neural network synchronicity may lead to the propagation and persistence of seizures. An abnormal FAA may indicate disruption in the synchronicity of frontal lobe neural networks, making it easier for seizures to spread to other brain regions, increasing the complexity of treatment and affecting prognosis.^[10,11]^ For example, some studies have found abnormal synchronized activity in the brain signals of epilepsy patients, which is associated with the severity and prognosis of seizures. FAA, as an indicator of frontal lobe neural electrical activity, may be related to this abnormal neural network synchronicity.^[12]^ Moreover, the developing brain in children exhibits high neural plasticity. SE may result in abnormal remodeling of brain neural networks, affecting neuronal connections, and function. FAA may be closely related to neural plasticity processes. An abnormal FAA may interfere with the normal developmental trajectory of the brain, leading to changes in synaptic plasticity. Synapses are critical structures for transmitting information between neurons, and changes in their plasticity may affect the balance between neuronal excitability and inhibition.^[13,14]^ In SE, abnormal synaptic plasticity may lead to abnormal strengthening or weakening of connections between neurons, increasing the brain’s sensitivity to seizures, reducing its resistance to seizures, and thus affecting patient prognosis. For instance, research has shown that synaptic structure and function in epilepsy patients undergo changes that are related to the frequency and prognosis of seizures. FAA may serve as an electrophysiological indicator of these changes in synaptic plasticity, reflecting abnormal brain development. Furthermore, neurogenesis refers to the process of neuron generation, which occurs to some extent in the brains of both children and adults. SE may impact the neurogenesis process.^[15]^ An abnormal FAA may be related to abnormal neurogenesis. Abnormal neurogenesis may lead to the abnormal construction of brain neural networks, affecting normal brain function. In SE, abnormal neurogenesis may make the brain more susceptible to seizures, impacting the brain’s ability to repair and adapt to seizures, thus affecting prognosis.^[16]^ Moreover, FAA may also be associated with the prognosis of pediatric SE by affecting signaling pathways related to neurogenesis, leading to abnormalities in neurogenesis and subsequently affecting prognosis.

Recent researches on pediatric SE have shown a close relationship between GFAP, S100-β protein, and GABA with the occurrence, progression, and prognosis of SE. Previous studies by Hong et al^[17]^ have also indicated the association of GFAP and GABA with SE, laying the foundation for further research. GFAP, a novel neural stem cell marker, has garnered significant attention in neurological disease studies, participating in the pathological processes of such conditions as stroke, epilepsy, and neurodegenerative disorders. S100-β protein, an acidic calcium-binding protein synthesized and secreted by glial cells, is a unique protein of the nervous system. Previous studies by Hanin A et al^[18]^ have shown that S100-β protein can serve as a diagnostic biomarker related to SE, providing a basis for the current study. Research suggests that levels of S100-β protein significantly increase in the blood or cerebrospinal fluid of patients with neurological disorders like brain injury, cerebrovascular diseases, and central nervous system inflammation. Therefore, this protein holds important diagnostic and prognostic value in these conditions, serving as a specific marker to reflect glial damage. Additionally, S100-β protein is involved in calcium-mediated signaling pathways, closely linked to the onset of epilepsy. In mammals, GABA acts as a vital inhibitory neurotransmitter in the central nervous system, playing essential roles in areas like the basal ganglia, thalamus, hippocampus, and cerebral cortex.^[19]^ Studies have also found^[20]^ that serum GFAP plays a crucial role in the process of epileptic seizures. Furthermore, neuroinflammation may lead to the breakdown of the blood–brain barrier, a crucial protective barrier shielding the brain from external harmful substances. When the blood–brain barrier is compromised, peripheral immune cells and inflammatory factors can enter the brain, exacerbating neuroinflammatory responses. Abnormal FAA levels may increase the permeability of the blood–brain barrier by affecting neuroinflammatory signaling pathways. The breakdown of the blood–brain barrier not only worsens neuroinflammation but also affects the efficacy of medications, making treatment more challenging and impacting the prognosis of pediatric SE.^[21]^ For instance, some studies have found that the function of the blood–brain barrier is impaired in epilepsy patients, correlating with the severity of seizures and poor prognosis. FAA may serve as an indirect indicator of neuroinflammation, reflecting the extent of blood–brain barrier disruption and subsequently affecting prognosis. Based on the further analysis of FAA and serum indicators, it is shown that posttreatment levels of GFAP, S100-β protein, and GABA in the FAA > 88.96 group are lower than those in the FAA ≤ 88.96 group, with statistically significant differences, further supporting the main findings of this study.

The results of this study suggest that FAA can play an application value in the clinical practice of status epilepticus in children. The FAA value was calculated through EEG measurement. When FAA > 88.96 (×10^‐3^ μV), it suggested that the patient had a high probability of a good prognosis and could be used as a reference indicator for prognosis assessment. Based on this, more precise treatment plans can be formulated clinically to strengthen monitoring and intervention for those with abnormal FAA. At the same time, combined with changes in serum indicators after treatment, FAA can also assist in judging the treatment effect, guide subsequent treatment adjustments, provide scientific basis for prognosis assessment and treatment decisions for children with SE, and improve the level of clinical diagnosis and treatment. Furthermore, although binary logistic regression analysis was performed in this study, the regression model may not have fully adjusted for all potential clinical confounding factors. Variables such as seizure etiology, seizure type, treatment protocol differences, and underlying neurological conditions were not comprehensively included in the adjusted model. Therefore, the predictive performance of FAA observed in this study should be interpreted cautiously. Future research should construct extended multivariable models incorporating key clinical covariates to improve the robustness and reliability of the findings.

While this study has achieved results, there are limitations that should be acknowledged. Firstly, the study is based on a single-center analysis, with relatively limited sample sources, which may not comprehensively represent the characteristics of pediatric SE patients in different regions and medical environments, thus limiting the generalizability of the research findings. Secondly, the retrospective study design used in this research may introduce information bias during data collection, such as incomplete medical records and inconsistent diagnostic criteria, potentially impacting the accuracy and reliability of the study results. Future researches should focus on conducting multicenter, prospective studies to further validate the predictive value of EEG-assessed FAA for the prognosis of pediatric SE. Due to the retrospective observational design of this study, causal relationships between FAA and clinical outcomes cannot be established. The findings should therefore be interpreted as associations rather than causal effects. Prospective studies with standardized data collection and controlled confounding factors are required to further validate the predictive role of FAA in pediatric SE. In addition, prognosis in this study was evaluated using the GOS assessed 1 month after discharge. Although this scale is widely used in neurological outcome assessment, it may not fully reflect the long-term neurological recovery and developmental outcomes in pediatric patients. Therefore, this outcome indicator may potentially underestimate or overestimate the risk of long-term prognosis. Future studies with longer follow-up periods are needed to obtain a more comprehensive evaluation of patient outcomes.

## 4. Conclusion

In conclusion, FAA demonstrates valuable predictive capabilities in the prognosis of pediatric SE, with mechanisms involving various aspects like neurophysiology, neural plasticity, neuroinflammation, and neurotransmitters. Future researches could delve deeper into exploring the combined application of FAA with other biomarkers and interventions targeting FAA abnormalities to provide more effective strategies for improving the prognosis of pediatric SE.

## Author contributions

**Conceptualization:** Liping Xie, Tao Yu, Jielan Zhou, Yaqin Zhang.

**Data curation:** Liping Xie, Tao Yu, Jielan Zhou, Yaqin Zhang.

**Formal analysis:** Liping Xie, Tao Yu, Jielan Zhou, Yaqin Zhang.

**Funding acquisition:** Liping Xie, Tao Yu.

**Investigation:** Tao Yu.

**Writing – original draft:** Liping Xie, Tao Yu.

**Writing – review & editing:** Liping Xie, Tao Yu.
